# IMPROVING ELDER MISTREATMENT SCREENING AND INTERVENTION IN DEMENTIA: EARLY NIA RESEARCH FINDINGS

**DOI:** 10.1093/geroni/igae098.2051

**Published:** 2024-12-31

**Authors:** Kristin Lees Haggerty, Terry Fulmer

**Affiliations:** Chair; Discussant

## Abstract

Elder mistreatment (EM) poses a significant public health concern for older adults, particularly those with mild cognitive impairment and Alzheimer’s Disease and Related Dementias (MCI/ADRD). This symposium presents innovative NIA-funded research aimed at assessing risk factors for EM and developing interventions to address these risks, focusing on caregivers and older adults with MCI/ADRD. The first presentation describes the development of a Screening, Brief Intervention, and Referral to Treatment model based on Medicare outpatient data and interviews with stakeholders. Preliminary findings highlight social determinants of health, caregiver stress, and education gaps as key contributors to EM risk. The second presentation introduces a caregiver risk assessment tool linked to an abuse prevention intervention for persons living with dementia (PLWD). The study demonstrates strong predictive capabilities in identifying modifiable caregiver risk factors associated with abuse. The third presentation describes adapting an evidence-based EM screening tool for home-based primary care providers. Qualitative data from seven sites inform the adaptation of the DETECT tool, addressing policy implications, barriers to recognition, and necessary adaptations for effective implementation. The fourth presentation highlights the development of a strategy to screen for caregiver neglect in PLWD. By evaluating existing tools and creating an item bank, this study aims to enhance measurement precision in elder mistreatment interventions. The final presentation reviews interventions targeting elder neglect by informal caregivers. Findings emphasize the need for feasible programs with rigorous evaluation strategies to address caregiver neglect effectively. This symposium presents projects aimed at improving EM risk assessment, identification, and interventions.

